# The Senior Editor of the Chicago Medical Journal Feeling Badly!

**Published:** 1858-09

**Authors:** 


### EDITORIAL.

### "*THE SENIOR EDITOR OF THE CHICAGO MEDICAL JOURNAL FEELING BADLY!*"

Under this *dignified caption*, the September number of the *Peninsular and Independent* contains three pages of editorial matter perfectly characteristic of the source from which they emanate. There are some men, so accustomed to act from motives purely personal and selfish, that, whenever they consider the doings of others, they almost instinctively lose sight of the acts themselves in an attempt to divine the motives which may have dictated them. Such men often see enemies where none exist, and are exceedingly valiant in *defending* institutions that have been assailed by no one.

In a former number of this journal we made some comments on the proceedings of the last annual meeting of the American Medical Association, in which we frankly stated that the business of the meeting was often embarrassed by the want of familiarity with parliamentary rules on the part of the President; that too much time was devoted to ethics; and that several special committees were treated unjustly by being entirely omitted from the proceedings. These statements, made solely for the purpose of advancing the permanent interests of the Association, by inducing more carefulness to avoid similar errors in future, Dr. Palmer gravely construes into an *attack* upon that organization. And "lest the *disparaging statements should wound the reputation of the Association*," he straightway takes upon himself the task of defence. But how does he perform it? Does he show one of our statements to be untrue or unjust? Oh no! nothing of the kind; but in place thereof, he gives the following *lucid* "explanation of our course." He says:

"Some of the reasons why the editor of the *Chicago Journal* received so unfavorable an impression of the meeting at Washington may be understood when it is stated, that, in the early years of the Association—in its infancy—*the which very bad things have convinced the editor that his reputed father assumed a large degree of parental* control over its affairs, but that since a more mature age has been attained by the offspring, and its independence of that attempted control has been manifested, the undutiful child is denounced, and the parental rod applied."

Now, reader, if you do not clearly comprehend the meaning of this paragraph, please read it again; and if you should still be somewhat in doubt, especially concerning the "reputed father," please ask the senior editor of the *Peninsular and Independant*, to give a *syntaxical* explanation of it. But he proceeds:

"Not to specify any possible high expectations of our neighbor unrealized at the Washington meeting, it cannot be improper to state what is intimated in the article under notice, that the author went to the meeting with a paper in his pocket—the continuance of his report on lactation, and its relations to pregnancy, etc.—and that, because he did not get an opportunity to read it, as he had read before in *full* the other instalments—or, at least, because the proceedings of the meeting were not conducted according to his fancy—he carried the paper away with him, and broke up the published series of his observations, not allowing this continuation to be published in the Transactions!"

Such is the substance, and very nearly the sum total, of what the editor of the *Peninsular and Independant* has "felt constrained to say in explanation" of our course, "lest our disparaging statements should wound the reputation of the Association," etc. The nonsense (we had like to have said *twaddle*) about the "reputed father"—"the offspring"—"the parental control"—"the possible high expectations," etc., is of a character well fitted for the columns of some small partisan country newspaper, but wholly unworthy the columns of a magazine devoted to the cultivation of a noble profession.

The only thing mentioned that has any relation to the doings of the Association, is the reading of a report which we had prepared in the performance of our duty as a special committee.

It will be seen by reference to his language, as quoted above, that the editor of the *Peninsular* fairly conveys to his readers the idea that we sought an opportunity to read our report *in full;* and because we could not do it, took offence and kept the paper. A statement so utterly devoid of truth should cause a blush to mantle the cheek, even of the senior editor of the *Peninsular and Independent.* The facts were precisely as follows: When about two-thirds of the special committees had been called and their reports disposed of, our report was duly announced by the President as next in order. We immediately rose to respond, holding half a sheet of letter paper in our hand. While thus on the floor, but before we had read a word, some member of the Association moved that all the remaining special committees be called in order, their reports read by their titles only, and referred to the committee of publication. We stated that the reading of the abstract we had prepared would not occupy more than from five to eight minutes of time. But the vote on the motion was taken immediately and carried. The President, however, instead of proceeding to call the remaining committees and have the titles to their reports read, entertained other business, and thus left four or five special committees without so much as a question whether they had prepared reports or not. Perhaps Dr. Palmer will regard this specimen of the mode of doing business as sufficient proof that the President was familiar with parliamentary rules. Be this as it may, however, it resulted in an entire omission of several special committees from the official records of the annual meeting of the Association. In our former article we complained of this as an act of injustice to the committees, and directly calculated to injure the Association by discouraging investigations on the part of committees. It may be that the editor of the *Peninsular Journal* is sufficiently anxious to see his name in print, to induce him to send a report to the committee of publication, when not so much as its title had been read to the Association, and not the slightest mention made of it in the official record of proceedings. If so, we have only to say that we neither partake of his anxiety nor approve of his ideas of propriety. In our former notice of the Washington meeting, we made no attack upon the Association or any of its members. As a true friend, ever anxious to insure its permanent prosperity, we pointed out such faults as we thought important, for no other purpose than to aid in preventing their repetition. But has it come to this, that every man who does not play the *courtier* and eulogize every item of the Association's proceedings and even the *manner* of conducting its business, must be published to the world as its enemy?

The able senior editor of the *North-American Medico-Chirurgical Review* ventures to criticise a paragraph in the late President's annual address, and straightway the *Nashville Journal* pours out upon him half a dozen columns of personal abuse. We venture to assert that devoting one-half of the annual meeting to mere ethics, and omitting all notice of several special committees, is not calculated to advance the permanent interests of the association; and the editor of the *Peninsular and Independent* proclaims that we have attacked that organization, racks his brains for some *possible personal* grief, and calls upon the medical public to apply to us "the contradiction and *rebuke*." We were about to ask who are the senior editors of the *Nashville* and *Peninsular* journals, and who constituted them *defenders* of the American Association? But we will not follow their example by so much as an allusion to personal motives or personal history. We are content to leave them to the judgment of an intelligent profession.

